# Orientation‐Dependent Phase Transformation Pathways Enabling Superior Superelastic and Elastocaloric Performance of NiTi Alloys

**DOI:** 10.1002/advs.202519606

**Published:** 2025-12-12

**Authors:** Jiaqi Lu, Muhammad Aziz, Hao Li, Chi Zhang, Zhifeng Huang, Fei Chen

**Affiliations:** ^1^ State Key Laboratory of Advanced Technology for Materials Synthesis and Processing Wuhan University of Technology Wuhan Hubei 430070 China; ^2^ Institute of Industrial Science The University of Tokyo Tokyo 153‐8505 Japan; ^3^ International School of Materials Science and Engineering Wuhan University of Technology Wuhan Hubei 430070 China

**Keywords:** additive manufacturing, elastocaloric effect, martensite variant microstructure, NiTi alloys, superelasticity

## Abstract

Developing elastocaloric materials that combine a large adiabatic temperature change with high superelastic stress and large recovery strain is crucial for the commercialization of solid‐state refrigeration. In this study, a scalable manufacturing route is introduced by integrating simulations with experiments to investigate the orientation‐dependent phase transformation behavior, producing NiTi alloys with performance surpassing that of all reported elastic metals in terms of superelasticity and elastocaloricity. Microstructural characterization confirmed that the preferred (001) grain orientation facilitates the generation of (001) compound twins in the [100](001) slip system, promoting the formation of low‐index reversible martensite and thereby enhancing the reversibility of the phase transformation. These results establish a direct link between crystallographic texture, variant selection, and functional performance, providing a scalable material solution for next‐generation solid‐state cooling devices.

## Graphical abstract

By integrating simulations and experiments, the preferred (001) crystallographic texture promotes reversible martensite formation through variant selection, enhancing both superelastic and elastocaloric performance. This work provides fundamental guidelines for designing high‐efficiency NiTi‐based functional materials.

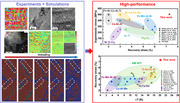

## 1 Introduction

Traditional vapor‐compression refrigeration technology suffers from low energy efficiency and relies on greenhouse gas refrigerants, making the development of zero‐emission, high‐efficiency solid‐state refrigeration technology imperative. Among various approaches, the elastocaloric effect is considered one of the most promising technical routes due to its significant advantages, such as simple driving methods and a high refrigeration capacity.^[^ ^1^ ^]^ In the pursuit of high‐performance elastocaloric materials, NiTi shape memory alloys (SMAs) exhibit large recoverable strains, high superelastic stresses (the critical stresses to induce long‐range martensitic transformations), and pronounced elastocaloric temperature change (*ΔT*), which are crucial for the commercialization of solid‐state refrigeration.

However, these functional requirements are challenging to achieve in a single material due to inherent trade‐offs among phase transformation stress, recovery strain, and thermal response. For instance, low‐superelasticity stress alloys can be easily deformed under low stresses but often exhibit a weak elastocaloric effect, which limits device sensitivity, accuracy, and thermal efficiency. Conversely, high‐superelastic stress alloys provide superior energy storage capacity and reduced material volume for a given strain. Yet maintaining large superelastic stress and *ΔT* simultaneously remains challenging.^[^ ^2^, ^3^ ^]^

Recently, introducing columnar grains with specific orientations has been effective in enhancing the functional properties,^[^ ^4^, ^5^ ^]^ and the texture exhibits a positive correlation with superelasticity in NiTi‐based SMAs. The stable melt pool and high thermal gradient in addictive manufacturing (AM) technology promote strong epitaxial growth, which is essential for achieving the dominant (001) crystallographic texture required by our design strategy. For example, the (100) texture can result in high recovery strains of 5.2% in Ni_50⋅8_Ti_49.2_ (at.%) SMAs by laser powder bed fusion (LPBF),^[^ ^6^, ^7^ ^]^ and numerical simulations also confirm that the NiTi alloys with the (100) texture present larger recovery strains.^[^ ^8^ ^]^ Compared with powder‐spreading printing technologies, such as LPBF and selective laser melting (SLM), laser‐directed energy deposition (L‐DED) technology can avoid excessive powder waste due to its powder‐feeding printing characteristics. Specifically, the grain morphology is influenced by temperature gradients and solidification rates, resulting in the formation of columnar and cellular grain zones at the center and edge of melt pools during the L‐DED process.^[^ ^7^, ^9^, ^10^ ^]^ Therefore, further research on the influence of grain structure on reversible phase transformation could be explored.

In fact, irreversible micro‐plastic deformation occurs inside the NiTi alloys because the movement of interfaces, such as phase interface, twin boundary, and variant boundary, induces extremely high local stress concentration in the surrounding austenite or martensite lattice during stress‐induced martensite transformation (SIMT) and reorientation of martensitic variants.^[^ ^11^ ^]^ In addition to the 12 correspondence variants (CVs), 24 habit plane variants (HPVs), and several B19′ twin modes are presented during SIMT,^[^ ^12^ ^]^ and the low‐index twins (such as (001) twins) are reversible.^[^ ^13^ ^]^ Under the stress field, those variants whose transformation strain (or twin direction) is most beneficial to adapt to the direction of external stress will be induced first. This means that at the beginning of SIMT, martensite with nearly single orientation is generated to promote the reversibility of the phase transition.^[^ ^12^ ^]^ However, micro‐control remains challenging, and thus, there is limited work on constructing the single martensite variant from an experimental perspective to enhance functionality.

On this ground, this work explores the multiscale mechanisms of the crystallographic orientation–dependent phase transformation behavior in NiTi SMAs and its implications for functional performance, aiming to fabricate NiTi alloys with a strong <001>‐orientated columnar grain structure by L‐DED technology, realizing a good combination of ultrahigh superelastic stress, large recovery strain, and elastocaloric effect. This work challenges this paradigm by demonstrating that crystallographic texture engineering offers a pathway to intrinsically enhance both the elastocaloric strength and the reversibility by revealing that a strong (001) orientation, thereby overcoming the conventional trade‐off and opening new pathways toward high‐performance solid‐state cooling and miniaturized functional systems.

## 2 Results

### 2.1 The Strong (001)‐Orientated Columnar Grain Structure

To improve the superelastic and elastocaloric performance, it is essential to prepare an ideal grain structure of L‐DED NiTi SMAs as closely as possible, namely, a strongly oriented columnar grain structure, which is primarily controlled by the temperature gradient and solidification front speed.^[^ ^14^ ^]^

**Figure 1:**
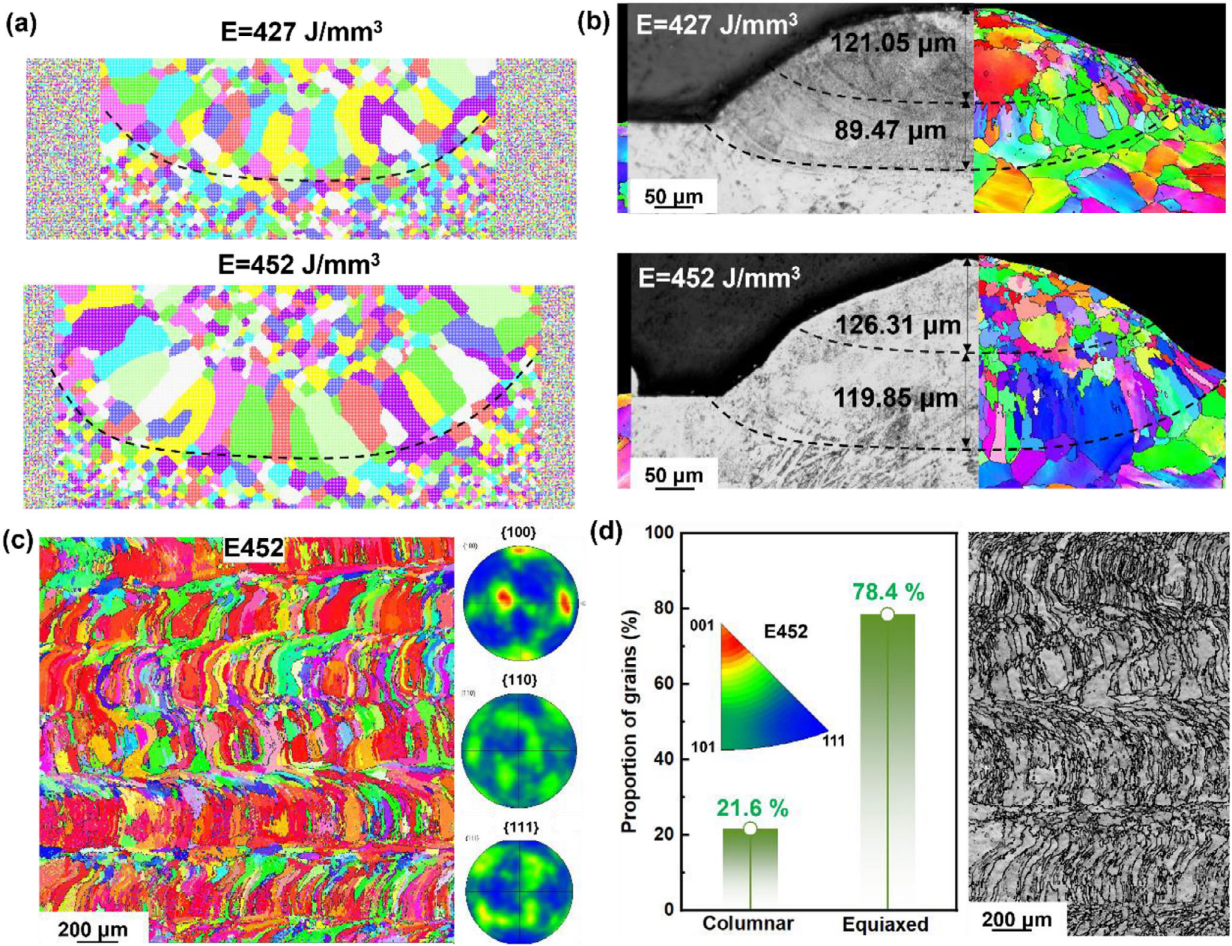
a) MD simulations showing grain evolution, and b) experimental results combining optical microscopy and EBSD analysis. At *E* of 427 J mm^−^^3^, the melt pool exhibits a shallower depth and narrower fusion zone with mixed equiaxed and columnar grains, whereas at 452 J mm^−^^3^, both melt pool depth and width increase, and columnar grains become dominant along the thermal gradient; c) The inverse pole figure (IPF) orientation, and corresponding pole figure maps of E452 sample, showing preferred orientation of <001>//BD. d) The grain morphology and the content of columnar grains in images of the two NiTi samples, prepared at *E* = 452 J m^−^^3^, illustrate that more columnar grains are produced with the larger *E*.

The molecular dynamics (MD) simulations reveal that an increase in laser energy density (*E*), calculated by *E* = *P*⋅*v* ^−1^⋅*h* ^−1^⋅*t* ^−1^,^[^ ^15^ ^]^ leads to a more pronounced melt pool depth and an evolution of grain morphology from relatively fine equiaxed grains to elongated columnar grains (**Figure** **1**a). These simulated results are in good agreement with the experimental observations (Figure 1b). The melt pool exhibits a shallower penetration depth (≈89.47 µm) and a relatively narrow fusion zone (≈121.05 µm), accompanied by a mixture of fine grains and partially developed columnar structures under a lower *E* of 427 J mm^−^^3^. In contrast, when the *E* increases to 452 J mm^−^^3^, the melt pool depth expands to ≈119.85 µm with a wider fusion zone, and the grain morphology is dominated by columnar structures aligned along the thermal gradient. These results highlight a strong correlation among laser energy input, melt pool geometry, and solidification texture, demonstrating that tailored *E* can effectively regulate the competitive growth between equiaxed and columnar grains during L‐DED.^[^ ^16^, ^17^, ^18^ ^]^ Therefore, combined with the results of mechanical properties (Figure S1, Supporting Information), the *E* values of 427–452 J m^−3^ are fabricated for exploration, namely E427, E440, and E452 samples. Unlike the other samples, the E452 sample shows a stronger <001> orientation (Figure 1c; Figure S2, Supporting Information), and most grains exhibit a preferred orientation of <001>//building direction (BD) in the Y‐Z plane, as indicated by the pole figure map (Figure 1c). With the increase of *E*, the content of columnar grains increases from 7.5 to 21.6% (Figure 1d; Figure S2, Supporting Information). Compared to the E452 sample, the E424 sample contains more equiaxed grains, as shown in Figure S3 (Supporting Information).

### 2.2 Superelastic Response and Elastocaloric Effect

**Figure 2:**
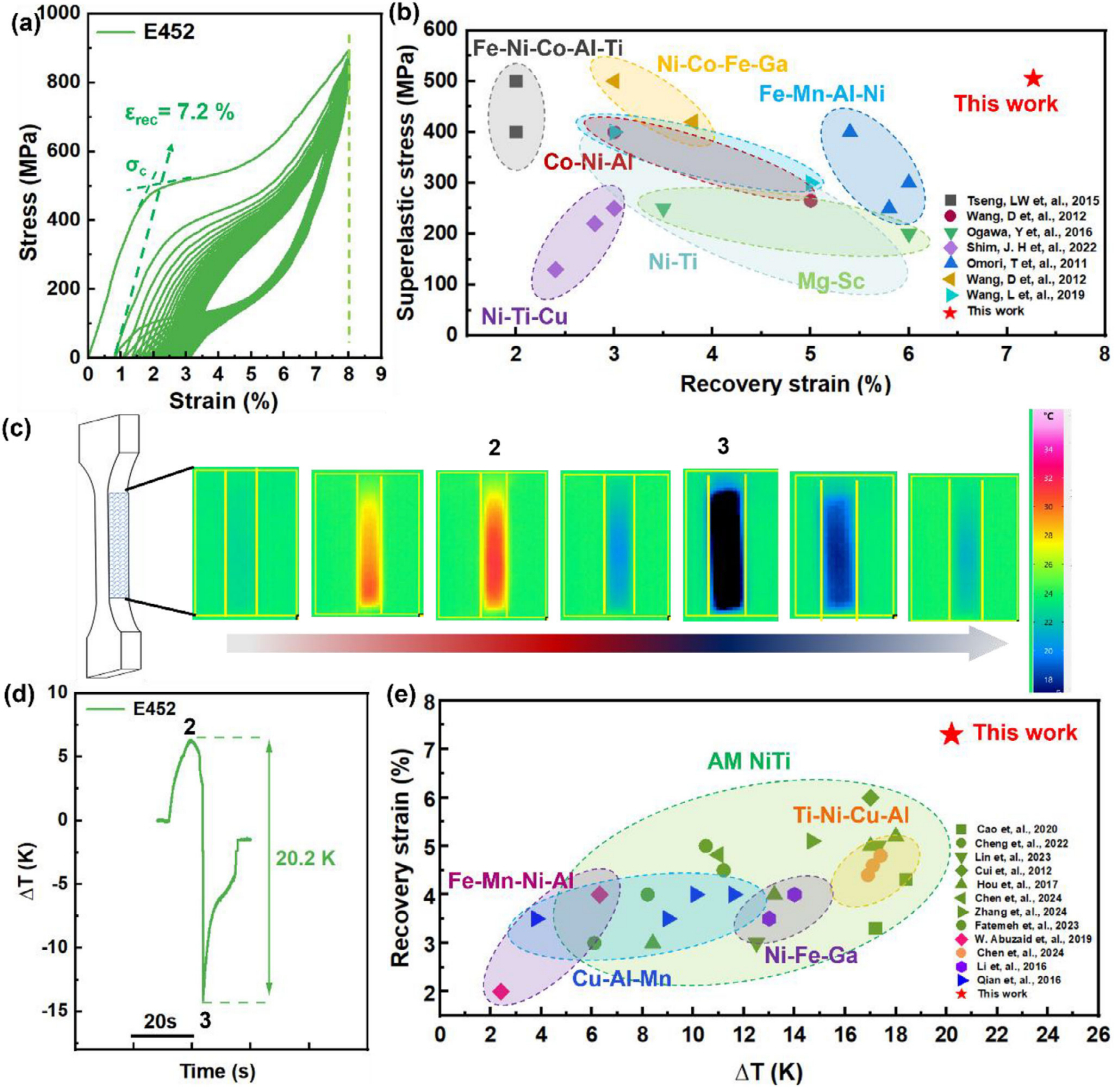
a) Cycling tensile curves of E452 samples, the superelastic stress (*σ*_C_) is determined by the tangent method and results of other samples are shown in Figure S4 (Supporting Information); b) The superelastic stress (*σ_C_*) vs the superelastic recovery strain (*ε_rec_*) of E452 sample and its comparison with conventional superelastic materials; c) Temperature change of the E452 sample during the loading and unloading process tested by infrared imager; d) the corresponding time‐*∆T* curve of E452 sample; e) Comparison of superelastic response and elastocaloric effect between E452 sample and other superelastic alloys, showing the synergistic improvement of recovery strain and elastocaloric effect of NiTi alloys in this work.

The superelastic responses of L‐DED NiTi alloys are illustrated in **Figures** **2**a and S4 (Supporting Information), where the positive effect of columnar grains on the recovery strain can be seen clearly. The E427 and E440 samples exhibit superelastic recovery strain (*ε_rec_*) of 3.0% and 5.0%, relatively narrow hysteresis loops, and low superelastic stress (*σ_c_*, which is the corresponding stress for the intersection point of two tangents in the loading stress‐strain curve, as shown in the inset of Figure 2a ^[^ ^19^ ^]^). By contrast, the E452 sample achieves both a high *σ_c_* of 500 MPa and a large ε_rec_ of 7.2% (Figure 2a), where the stress‐strain loops exhibit a larger hysteresis area, indicating a higher energy dissipation capacity and superior strain accommodation through extensive martensitic transformation. The trade‐off between high *σ_c_* and large *ε_rec_* is overcome in the present NiTi SMAs, which are superior to conventional superelastic alloys, such as Fe‐, Mg‐, Co‐, Ti‐Ni‐, Ni‐based, and recently‐found high‐entropy SMAs,^[^ ^20^, ^21^, ^22^, ^23^, ^24^, ^25^, ^26^, ^27^ ^]^ as shown in Figure 2b, providing potential applications for the basic material of the self‐expanding bracket, to achieve a large response by preloading components.^[^ ^28^, ^29^ ^]^ Importantly, the *ε_rec_* is not completely proportional to the columnar grain content. Excessive columnar grains, such as 30.1%, actually have an adverse effect on ε_rec_ and broke at the 16th cycle (Figure S5, Supporting Information), also reflecting the important role of (001) texture in enhancing the recovery strain.

A high recovery strain could make NiTi alloys promising candidates for energy absorption and storage applications, enabling solid‐state refrigeration instead of the traditional vapor‐compression system, which has the advantage of environmental protection. The E452 sample with an *ε_rec_* of 7.2% has incomparable advantages in the elastocaloric effect (Figures 2c,d). Specifically, the martensitic transformation occurs during the stress process, and the temperature rises by ≈7 K (point 2 in Figure 2c) due to an exothermic process, reaching a strain of 8%. Subsequently, the stress is unloaded, and the martensite transforms into austenite, an endothermic process that occurs as the temperature drops by ≈13 K (point 3 in Figure 2c). Consequently, the temperature difference is as high as 20.2 K in the whole process, which not only exceeds other superelastic alloys but also is the best record for additively manufactured (AMed) NiTi alloys, as presented in Figure 2d.^[^ ^4^, ^5^, ^20^, ^28^, ^30^, ^31^, ^32^, ^33^, ^34^, ^35^, ^36^, ^37^, ^38^, ^39^, ^40^, ^41^, ^42^ ^]^ This is remarkable, as no AMed NiTi alloys have previously shown synergistic improvements in recovery strain and the elastocaloric effect. It should be emphasized that the fatigue performance of our current sample needs improvement, which is an essential focus for our future work while preserving the high recoverable strain and strong elastocaloric response.

### 2.3 Martensite Variant Microstructures Evolving in Loading

**Figure 3:**
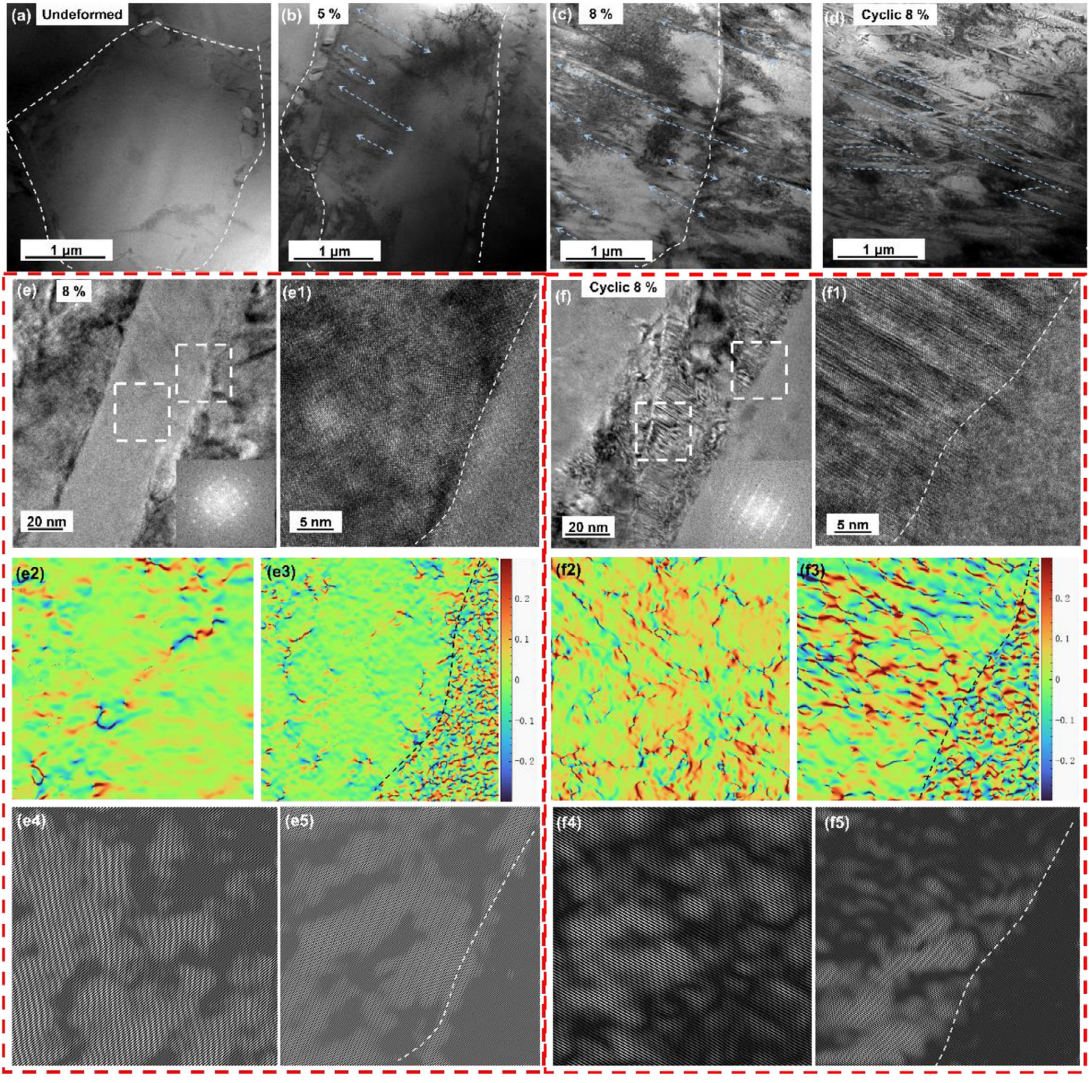
Structural characterization for the E452 NiTi alloy under different stress states (//BD). Bright‐field TEM images of a) undeformed, b) 5%, c) 8%, and d) cyclic 8%; Magnified bright‐field image under e) 8% and f) cyclic 8%, and insets in (e and f) are the corresponding SAED patterns; High‐resolution TEM (HR‐TEM) image to display the interface between the B2 austenite and B19` under the (e1) 8% and cyclic (f1) 8%; Geometric phase analysis (GPA) maps of strain components in (e2) the B19`and (e3) the interface corresponding to (e), in (f2) the B19`and (f3) the interface corresponding to (f); the IFFT pattern of strain components in (e4) the B19`and (e5) the interface corresponding to (e), in (f4) the B19`and (f5) the interface corresponding to (f).

Then, what are the differences in the E452 sample in the phase transition process due to the strong (001) grain texture? The structural evolution of martensite variants from the undeformed state to after 30 cyclic loading (pre‐strain of 8%) in the E452 sample is further explored in **Figures** **3**a–d, and the most martensite variants propagate from the grain boundary (GB) in the same direction (Figure 3b), and cross through the GB during cyclic loading (Figure 3c). From the high‐resolution image in Figure 3e, it can be seen that the interface between martensite and the B2 matrix exhibits a uniform transformation strain of ≈3%. (Figures 3e1,e3), and there are fewer dislocations in this martensite (Figures 3e4,e5). Moreover, dislocations and high transformation strain are observed in the elongated martensite after cycling (Figures 3f‐f3), thereby alleviating the strain difference. Interestingly, another shorter martensite variant appears after cyclic tensile in Figure 3d, and thus wedge‐shaped microstructures are shown due to the suitable applied stress state, which is regarded as the self‐adaptation of martensite during deformation. In the meantime, the transmission electron microscopy (TEM) micrographs of 16 different regions of the E452 sample after cycle tensile are shown in Figure S6 (Supporting Information), confirming the preferential formation of specific martensite variants.

**Figure 4:**
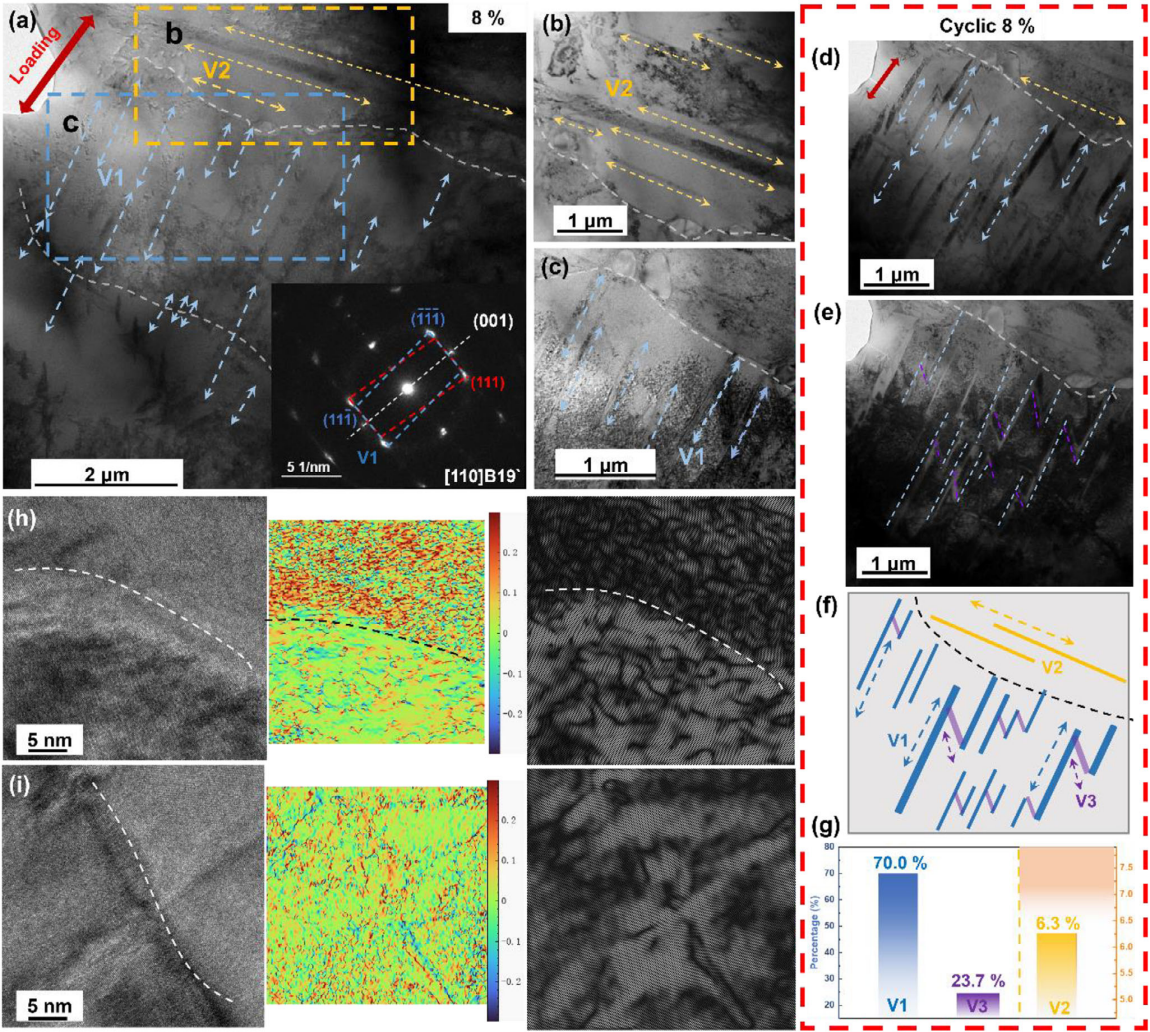
a) Martensite variant microstructures under strain of the 8% in E452 samples; The enlarged view of V2 b) and V1 variants c) in (a); d) The V1 microstructures after cyclic loading and e) the dark field image showing that the V3 variants; f) Schematic diagram of the distribution and g) proportion of the three variants in the matrix (according to Figure S6, Supporting Information); h) HR‐TEM images of grain boundary that prevents martensite from growing, and its strain distribution diagram; i) HR‐TEM images of grain boundary through which martensite cross, and its strain distribution diagram.

**Figure** **4**a further reveals the distribution and structure of different martensites. Martensite variants V1 and V2 are observed in three adjacent grains, and their direction is perpendicular. It is found that V1 cannot cross the grain with the V2 variant. The selected area electron diffraction (SAED) pattern corresponds to B19` lattices in (001) compound twin relation (inset of Figure 4a). These twin boundaries serve as effective accommodation mechanisms for transformation strains, minimizing defect accumulation and maintaining structural integrity during repeated loading‐unloading cycles. The two variants are more clearly visible after changing the ribbon axis direction in Figures 4b,c. After cyclic loading, the third variant (V3) can be identified (Figure 4d), and the dark‐field (DF) image (Figure 4e) shows the V1 and V3 variants. The distribution of the three variants in the matrix is shown in Figure 4f. Statistics of martensite variants in 16 different regions (Figure S6, Supporting Information) show that V1 accounts for ≈70%, while V2 and V3 account for only a small proportion (Figure 4g). A small number of V3 variants were observed in bright‐field (BF) images at different dip angles (Figure S6, Supporting Information), indicating that these laths originate from the same parent phase orientation, which is consistent with the unified variant selection mechanism controlled by B2 orientation and can provide local strain coordination. It should be emphasized that the E452 sample does not contain only these three variants, and other variants are too small to be counted.

Notley, two modes of martensite facing the GB constraint are directly observed in Figure 4a. First, martensite changes direction at the GB, and other variants with different orientations are formed in the adjacent grain. The deformation and strain differences at this interface are larger, as shown in the GPA map in Figure 4h. Second, the martensite lath directly crosses the GB, indicating that adjacent grains have good coordination in the region with the same texture, leading to a smaller strain difference at the interface and fewer dislocations, as shown in Figure 4i.

## 3 Discussion

Next, the orientation‐dependent transformation mechanism underlying the exceptional superelastic response and elastocaloric effect in the present E452 sample is described as follows.

First, the inherent crystallographic characteristics of the (001) orientation are focused on. The B2 austenite of NiTi alloys exhibits pronounced elastic anisotropy. According to commonly reported single‐crystal elastic constants, the Young's modulus along the (001) direction is significantly lower than that along the (110) and (111) directions,^[^ ^34^, ^43^ ^]^ indicating that the (001) orientation behaves as an elastically softer direction. Moreover, the atomic arrangement of the B2 structure varies among different crystallographic planes. The {100} planes, corresponding to the (001) texture, possess a distinct in‐plane atomic configuration and a relatively lower atomic planar density compared with {110} and {111} planes.^[^ ^44^ ^]^ Consequently, under the same stress, the strain energy changes, corresponding to different grain orientations. Elastic soft direction (such as (100)) will produce greater strain or favorable strain decomposition, causing the driving force direction to be dependent, and then affect the nucleation conditions and nuclear barrier.^[^ ^44^, ^45^, ^46^ ^]^

**Figure 5:**
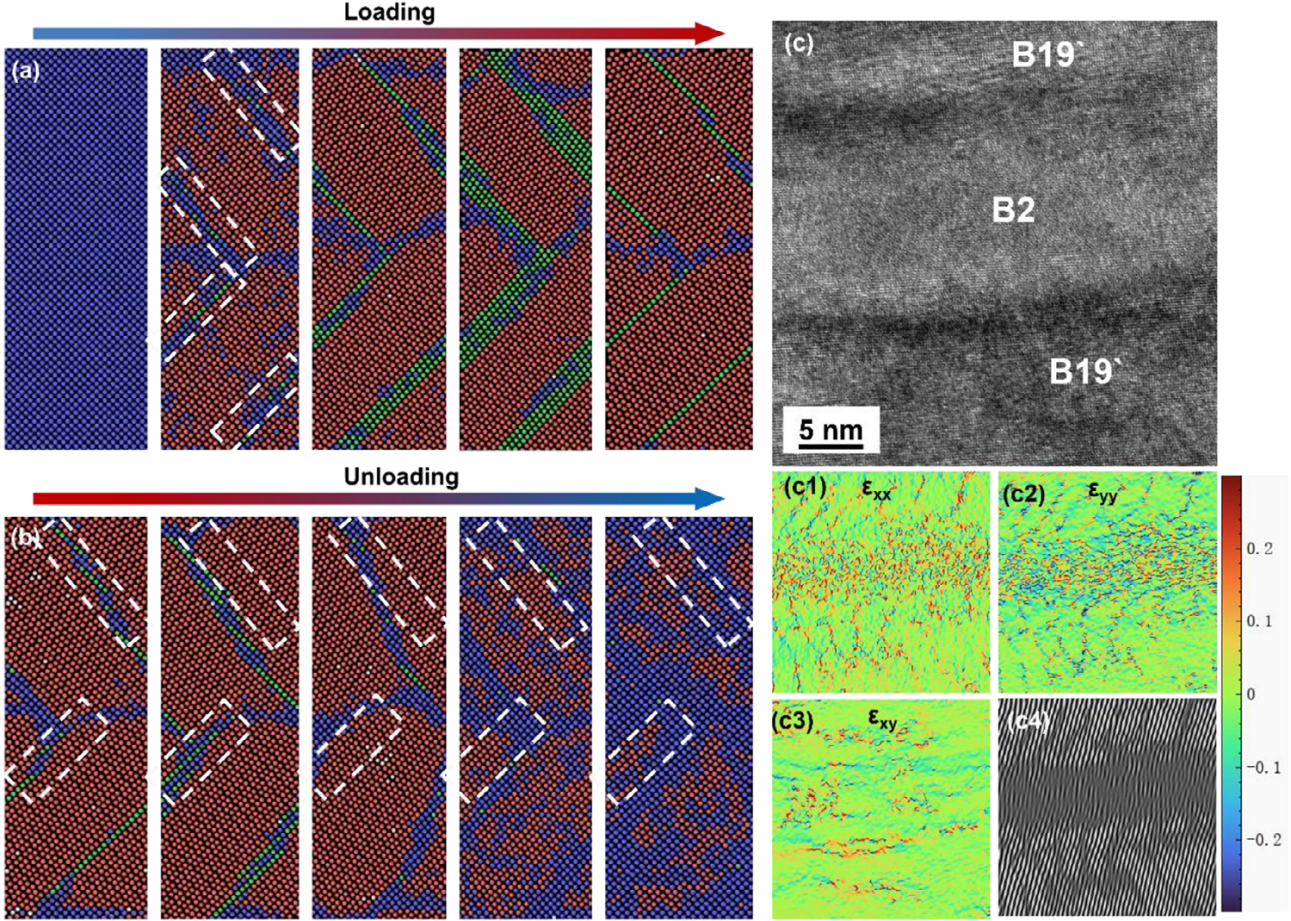
a) MD simulations: the superelastic process of Ni‐Ti single crystal with (001) grain orientation during the loading‐unloading process. The initial state is B2 (blue), and B2 is transformed into B19 (red) after loading, but FCC (green) is produced in a specific area (white dotted dox), where B2 preferentially grows during unloading; b) the HRTEM image and c) the GPA results of the interface between B19` and B2 in the E452 sample, showing the there are more stress and dislocations in B2` area but local stress concertation (strain difference) is small, illustrating the FCC phase could relax the interfacial strain.

MD simulation results indicate that the face centered cubic (FCC) phase (green), a strain‐buffered metastable phase formed in the high‐strain local region to achieve phase transformation compatibility, appears at a specific position during loading, exhibiting a herringbone distribution. Even if martensite (red) forms in large quantities, the FCC phase still remains at a specific interface position, forming a typical layered phase transition structure (**Figure** **5**a). During unloading, B2 is preferentially produced at the high‐strain FCC region (indicated by the white dotted box in Figure 5b), which is beneficial for uniform deformation. The appearance of FCC can relax the interfacial strain more effectively and provide a more favorable phase transition path (B2‐FCC‐B19`), thus reducing the total energy of the system. This corresponds to the microstructure. High‐resolution TEM (HRTEM) reveals that strain contrast is observed across the indicated interface between B2 and B19` of the E452 sample (Figure 5c), suggesting the presence of localized lattice distortion and strain accumulation (Figures 5c1–c3),^[^ ^47^ ^]^ which is obviously lower than the strain difference between the other two variants and the matrix (Figure S7, Supporting Information), where the strong tensile strain is observed in V3 variants; that is, the local stress concentration at the interface caused by phase transformation is alleviated. Also, consistent with the results in Figure 3f3, dislocations are observed in martensite variants. Furthermore, some previous works have confirmed that NiTi alloy with near (001) orientation will form a group of specific and limited martensite variants during loading, and different structural regions may appear in these different martensite variants, which are derived from different twin systems in B19' martensite, representing strain‐coordinated domains.^[^ ^48^, ^49^, ^50^ ^]^ At the same time, the experimental results illustrated that SIMT was carried out catalytically in AMed NiTi alloys with a <001>//BD texture,^[^ ^47^ ^]^ further demonstrating that grains with a (001) orientation are more beneficial for SIMT. The lattice distortion tensor of <001> corresponds to the principal axis direction of the cubic cell, which is orthogonal to the coordinate system, and strain coordination can be completed with only a few variants in the phase transformation process.^[^ ^51^, ^52^ ^]^ Hence, variant selection is simple, and there is a smooth transition. In contrast, the (011) orientation corresponds to the plane diagonal direction of the cubic cell,^[^ ^53^ ^]^ and the stress direction is not completely consistent with the main axis of martensite deformation, which requires the cooperation of multiple martensite variants to achieve strain compatibility.

Second, driven by external forces, martensitic transformation is not random,^[^ ^51^ ^]^ and the preferential formation and growth of martensitic variants depend on the mechanical driving force, which is related to the stress tensor and the corresponding transformation strain tensor. Only those variants with significant positive transformation strain projection in the tensile direction can obtain a sufficient driving force and occupy a dominant position. In detail, each variant (*i*) corresponds to a transformed strain tensor (*ε_i_*), and the mechanical driving force *W_i_* ≈ *ε_i_* ×*σ* for variant i is exerted under uniaxial tension (*σ*).^[^ ^54^, ^55^ ^]^ Only variants with a positive, large transformation strain projection in the loading direction can achieve the maximum *W_i_* and form preferentially during SIMT. The groups that can obtain high Wi differ, leading to the simultaneous occurrence of multiple variations to meet the local stress/displacement compatibility in the sample. The (001) orientation has the largest *W* through the uniform orientation. Therefore, for the E452 sample, most grain transformation driving directions are the same, thus avoiding this multi‐directional competition. In fact, when the martensitic transformation occurs, multiple shear systems are activated by grains with different orientations at the same time, resulting in a variety of martensitic variants. Different B19' variants require different twin planes to coordinate, and they interact with each other at grain boundaries or within grains,^[^ ^12^, ^52^ ^]^ resulting in different transformation strain directions and local stress concentrations. To alleviate this, the matrix needs to stimulate more secondary variants to adapt again, as depicted in Figures 3d and 4a. The wedge structures consist of two V1 variants and a V3 variant that coordinates the strain.^[^ ^56^, ^57^ ^]^ This exquisite arrangement is a crystallographic proof that martensitic transformation can minimize energy. It shows that the phase transition process remains controlled and crystallographically coordinated even after cyclic loading, proving that the strain system realizes overall strain coordination through local optimal selection.^[^ ^57^, ^58^ ^]^ Also, tiny martensite variants that are newly formed at the grain boundaries that hinder the propagation of martensite are observed in Figures S8a,b (Supporting Information). Influenced by the V1 variant, these variants do not grow along the GB. In addition, V1 martensite can achieve strain coordination through local slip and interface relaxation with the B2 matrix. As shown in Figure 3f, the many dislocations in the martensite lath provide evidence that martensite realizes lattice invariant shear through local slip. Figure S8c,d (Supporting Information) also confirms that there are dislocations in the B2 matrix between two martensites, which proves to relax the interfacial strain and promote the reversibility of phase transformation. In addition, equiaxed grains have more high‐angle grain boundaries, and local stress concentration and compatibility requirements promote the diversification and twinning of variants,^[^ ^19^, ^38^ ^]^ allowing them to adapt to the strain field on the microscale. And it is confirmed by the presence of multiple B19` diffraction peaks in the XRD pattern after cycling in the E427 sample, indicating an increased complexity and heterogeneity of martensitic transformation after loading, instead of the low‐index martensite peaks in (Figure S9, Supporting Information), only including (020) B19' and (002) B19' reflections.

**Figure 6:**
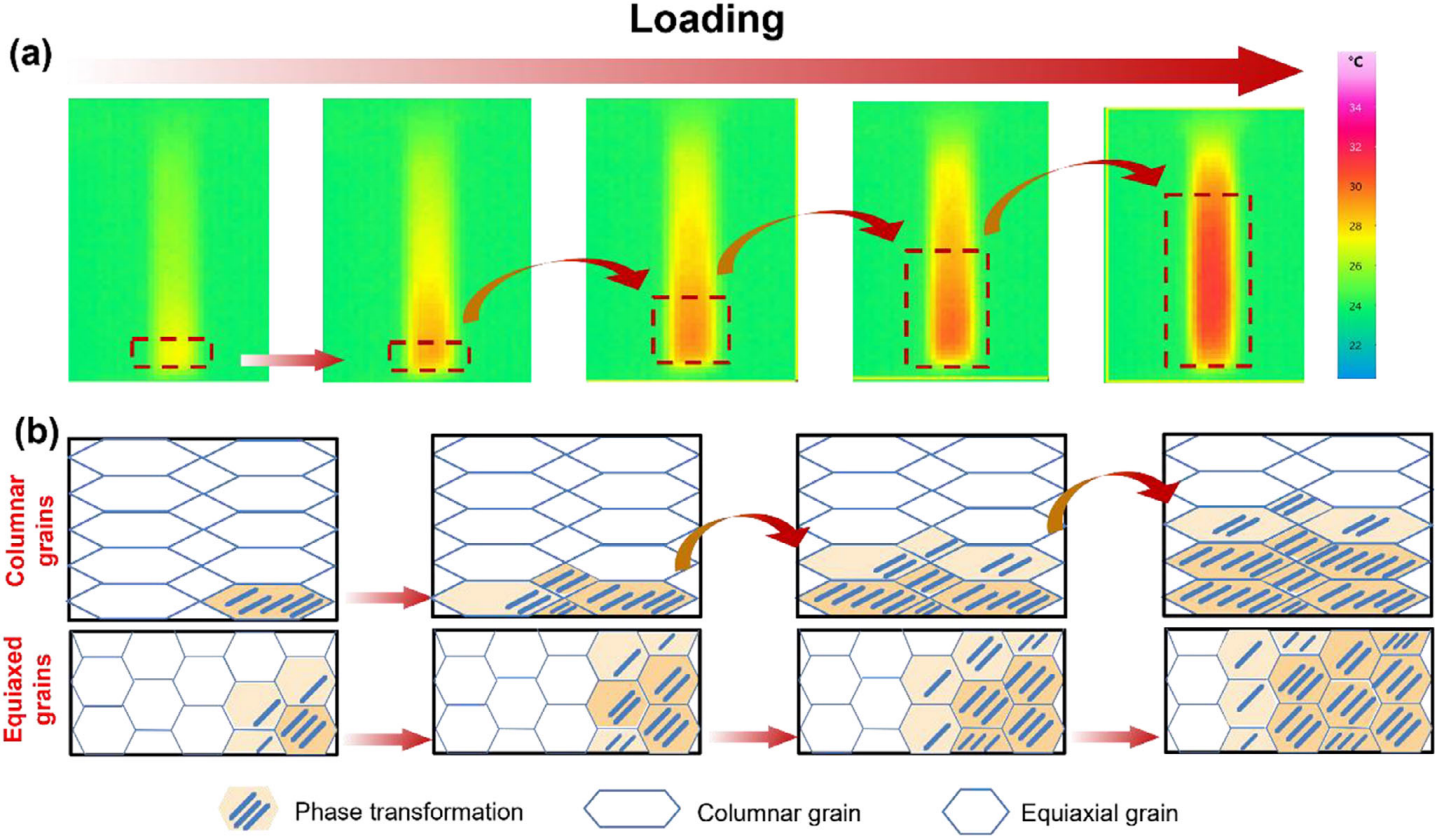
a) The temperature change of E452 samples, showing the regular and uniform deformation during loading; b) the schematic diagram of martensite transformation in equiaxed grains and columnar grains, respectively, during the loading process.

Third, columnar grains also have advantages in phase transformation. The temperature distribution of the E452 sample is revealed during mechanical loading (Figure 6a). The Lüders‐type deformation mechanisms in martensitic and austenitic alloys are likely similar in nature.^[^ ^59^ ^]^ Reasonably, the columnar grains and equiaxial grains show different phase transformation behaviors (Figure 6b). The martensite tends to propagate along the long axis of grains where there is no GB hindrance, where a favorable variant can extend along the long range of columnar grains, forming a relatively continuous phase transformation zone in columnar grains. However, the phase transformation exhibits a random mode in equiaxed grains due to the hindrance of grain boundaries. As illustrated in Figure 4h,i, one variant can cross the grain boundaries, reducing the energy dissipation at the grain boundaries. However, due to the large difference in grain orientation, martensite changes direction, resulting in a large strain field at the GB, indicating that polycrystalline materials adapt to the strain coordination mechanism of GB constraints by activating different but optimal variants. During the unloading process, B2 preferentially nucleates in the high‐strain‐energy region near the defects in the grain rather than the (001)M compound twin boundaries, showing a uniform phase transition trend, and propagates along the junction plane of B19` variants instead of the boundary of (001) compound twins.^[^ ^12^, ^60^, ^61^, ^62^ ^]^ Hence, the temperature field gradually returns to a uniform distribution (Figure S10, Supporting Information), reflecting the reversible superelastic deformation of NiTi alloys in **Figure** **6**.

Lastly, the strain compatibility of (001) compound twins takes precedence in this regard. This relates to the slip system during the martensite transformation. The atomic structures of the B2 austenite and B19` martensite are visualized as “cells” built by the software CrystalMaker, as depicted in Figure S11 (Supporting Information). The monoclinic martensite lattice is sheared to the left or right due to monoclinic distortion, resulting in martensite variants V1 and V2 originating from a single (1‐10) austenite plane (Figure S11a, Supporting Information). Because the Ti─Ti bond is weak (indicated by the blue dotted line),^[^ ^12^, ^63^, ^64^ ^]^ the monoclinic structure is easy to shear in the (001) plane along the [100] direction, so that (001) compound twins are formed between variants V1 and V2 under low stress, and dislocation slip in the [100](001) direction is realized.^[^ ^65^ ^]^ In particular, the detwinning of (001) compound twins is considered to proceed via slip of partial dislocations in the [100](001) slip system under high stress (Figure S11b, Supporting Information). Fortunately, the (001) plane is the lattice plane with the largest lattice spacing and the highest density in the B19' monoclinic structure. Therefore, compared to other slip systems, the critical stress for activating the [100](001) dislocation slip system is very low.^[^ ^8^, ^65^ ^]^ The monoclinic lattice on the (001) plane sheared in the [100] direction can respond by (001) compound twinning. Furthermore, the (001) plane is a twin plane with low interface energy and good strain coordination, which can minimize interface mismatch energy, improve interface compatibility (Figure 5c; Figure S9, Supporting Information), reduce energy dissipation during phase transformation, and promote uniform deformation.

In summary, we demonstrated a multiscale design strategy that combines simulations with L‐DED to provide direct evidence that crystallographic texture can be deliberately engineered to enhance both superelastic and elastocaloric performance in NiTi alloys. The results show that the NiTi alloy with a strong (001)‐orientated columnar grain structure not only possesses high superelastic stress and large elastic recovery strain beyond the previously reported AMed NiTi SMAs, but also has an incomparable elastocaloric effect among all elastic metal materials. This outstanding performance is attributed to the (001)‐oriented columnar grain, which promotes the formation of single reversible martensite variants, together with the coordination of phase transformation strain near GBs and martensite, and (001) compound twins during SIMT, thereby enhancing the reversibility of the phase transformation. Our findings reveal the atomic‐scale mechanisms of orientation‐dependent transformation, thereby uncovering the untapped advantages of AM technology in producing a specific grain structure. This study also provides a scalable route for the development of solid‐state cooling materials, bridging the gap between fundamental understanding and practical applications.

## 4 Experimental Section

### Sample Preparation

The commercial NiTi SMA powders with a particle size of 53–106 µm exhibited a high size uniformity in this work, as shown in Figure S12a (Supporting Information), and were produced by the electrode induction‐melting gas atomization technique (AVIMETAL AM Powders Co., Ltd., Beijing). Bulk NiTi SMAs were manufactured (40 × 20 × 10 mm^3^) using a commercial L‐DED machine (LENS‐150) equipped with a fiber laser with a hatch spacing value (*h*) of 0.381 mm, laser power (*P*) of 330–380 W, scanning speed (*v*) of 20 inch/min, layer thickness (*t*) of 0.254 mm, and rotation angle *φ* of 90° (Figure S12b, Supporting Information) in this work.

### Superelasticity and Elastic‐Thermal Testing

The LDED NiTi samples were removed from the building plate for further exploration via wire electrical discharge machining. Utilizing a universal electronic testing system equipped with an extensometer (BM320‐H, Baoma, China, 10 kN), cyclic tensile tests were conducted at 300 K. The tests involved loading the stress from 0 MPa to 8% and then unloading it to 5 MPa for 30 cycles at a rate of 4 × 10^−3^ s^−1^. The test samples were cut into dog‐bone‐shaped specimens, whose gauge sizes are shown in Figure S9c (Supporting Information). During the superplastic experiments, a thermal and infrared imager was used to conduct in situ observation of the specimen.

### Characterization of Phase Transition

The surface roughness was effectively reduced by mechanical polishing for further tests. Differential scanning calorimetry (DSC, STA 449 F3 Jupiter, Germany) was used to determine the phase transition temperature at heating and cooling rates of 10 °C min^−1^, with a test temperature range of −80 to 120 °C under a nitrogen atmosphere. To investigate the phase change before and after loading, an X‐ray diffraction (XRD) test using Cu Kα radiation was conducted.

### Microstructural Examination

After polishing samples with SiC grit papers and etching in solution (HF: HNO_3_: H_2_O = 1:3:6) for 10 s, optical microscopy (OM) and scanning electron microscope (SEM, JSM‐7500F, Japan) were selected to observe molten pool and grain morphologies, respectively. The grain orientation and microstructures were also characterized using electron backscattered diffraction (EBSD, C‐nano, Oxford, USA) and analyzed with Channel 5 software. The samples were initially mechanically ground and then electropolished in a sulfuric acid‐methanol solution at room temperature with a 30 V voltage. The microstructural evolution, including B19`, twins, grains, and dislocations, was examined in more detail using TEM (Talos F200X, FEI, USA). TEM foils with a diameter of 3 mm in the cross‐section (parallel to the BD) and 0.3 mm in thickness were wire‐cut from the ROI in each tensile sample, and mechanically polished to a thickness of <50 µm. Finally, the center of each foil was perforated using a GATAN695 Ar‐ion thinning instrument. Different imaging modes (e.g., bright and dark fields) were employed to investigate the phase structure, twin and dislocation characteristics, with the help of DigitalMicrograph and Strain software.

### Simulation Approaches

Grain evolution is modeled using a Monte Carlo model. The model is available as an application in SPPARKS^[^ ^66^ ^]^ and has been described in detail elsewhere.^[^ ^67^, ^68^, ^69^ ^]^ Here, we provide a brief overview of the essential components of the method. This model uses an ellipsoid to describe the geometric shape of the melt pool. The model simulates grain growth by attempting to flip a site, with the flipping probability controlled by the mobility *M(T)* and the Arrhenius equation:

(1) $$P=\left\{\begin{array}{cc} M\left(T\right) & \;\Delta E\leq 0,\\ M\left(T\right)e^{-\frac{\Delta E}{kT}} & \;\Delta E>0. \end{array}\right.$$

Whether a flip is accepted is determined by energy and mobility considerations. For instance, even if a flip reduces the energy, it may be rejected if it occurs in a region far from the melt pool. In contrast, near the melt pool, where atomic activity is high and GB migration is facile, flips are readily accepted.

All MD simulations were performed using the LAMMPS package. The interatomic interactions in the NiTi alloy were described using the MEAM potential developed by Won‐Seok Ko et al.,^[^ ^70^ ^]^ which has been widely used to simulate phase transformations in NiTi alloys.^[^ ^58^, ^71^ ^]^ Periodic boundary conditions were applied throughout the simulations. The samples were first subjected to energy minimization. Following this, they were relaxed to a stable state at 300 K under NPT ensembles before the tensile process. The *z*‐axis is set as the stretching direction, and pressure is controlled in the *x* and *y* directions during the stretching process, with a tensile strain rate of 5 × 10^8^. The simulation results were visualized using OVITO.^[^ ^72^ ^]^

## Conflict of Interest

The authors declare no conflict of interest

## Supporting information



Supporting Information

## Data Availability

The data that support the findings of this study are available from the corresponding author upon reasonable request.
