# Supplementary material for: Orientation‐Dependent Phase Transformation Pathways Enabling Superior Superelastic and Elastocaloric Performance of NiTi Alloys
Source: Adv Sci (Weinh). 2025 Dec 12;13(12):e19606. doi: 10.1002/advs.202519606 (PMC12948238; doi:10.1002/advs.202519606)
Supplement: Supplementary file 1 — Supporting Information [file ADVS-13-e19606-s001.docx]

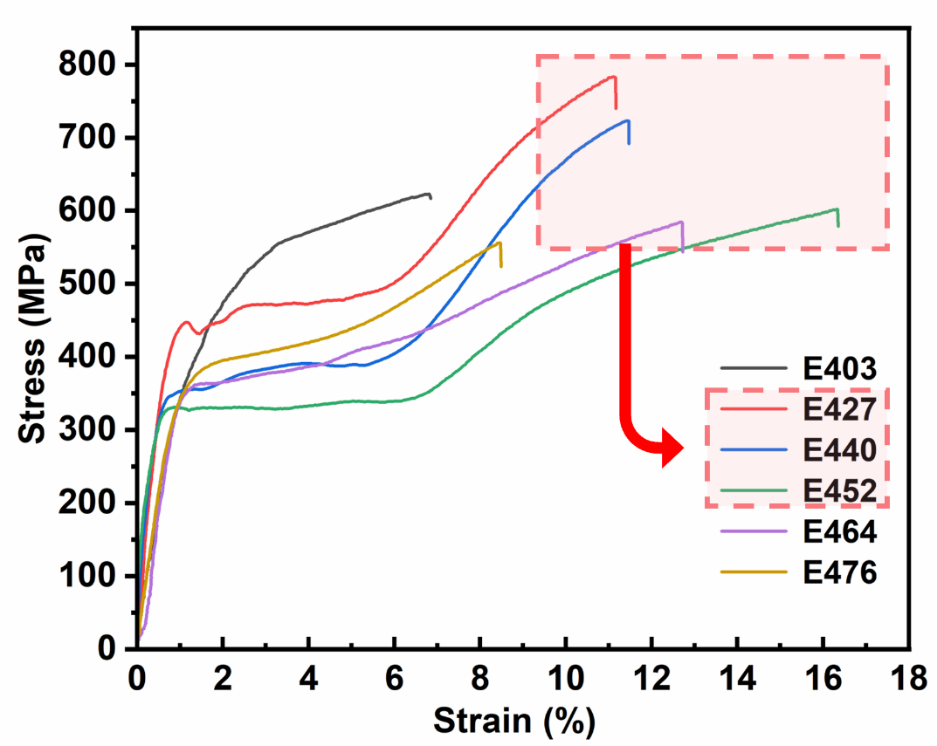


**Fig. S1** The mechanical properties of L-DED NiTi SMAs fabricated from E of 403 to 476 J/m^3^, illustrating that the elongation of the alloy with too low or high E is low, such as NiTi SMAs with E of 403 and 476 J/m^3^, but the strength of the alloy decreases with the increase of E, such as NiTi SMAs with E of 440 and 452 J/m^3^. Therefore, the alloys with E in a suitable range from 427 to 452 J/m^3^ are selected for research.


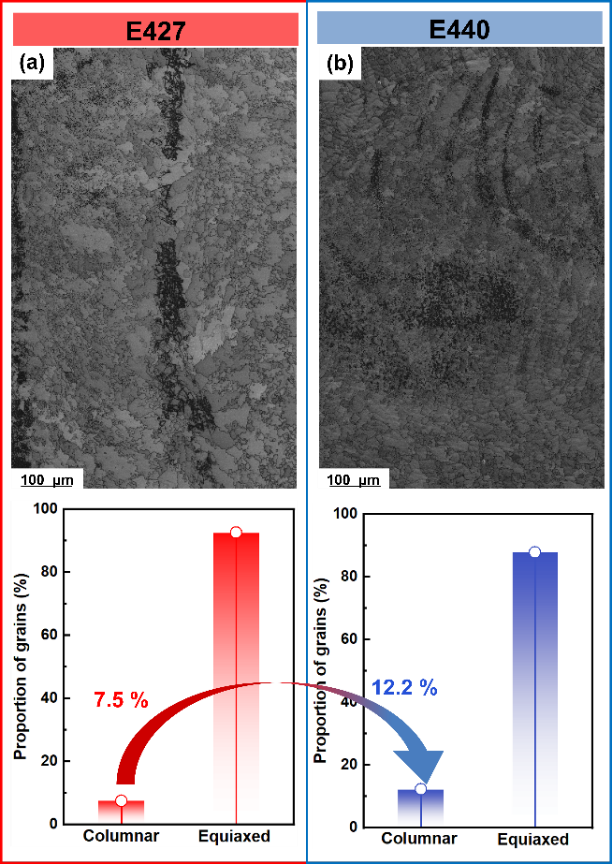


**Fig. S2** The grain morphology and the content of columnar grains images of the two NiTi samples prepared by E=452 J/m^3^, illustrating that more columnar grains are produced with the larger E.


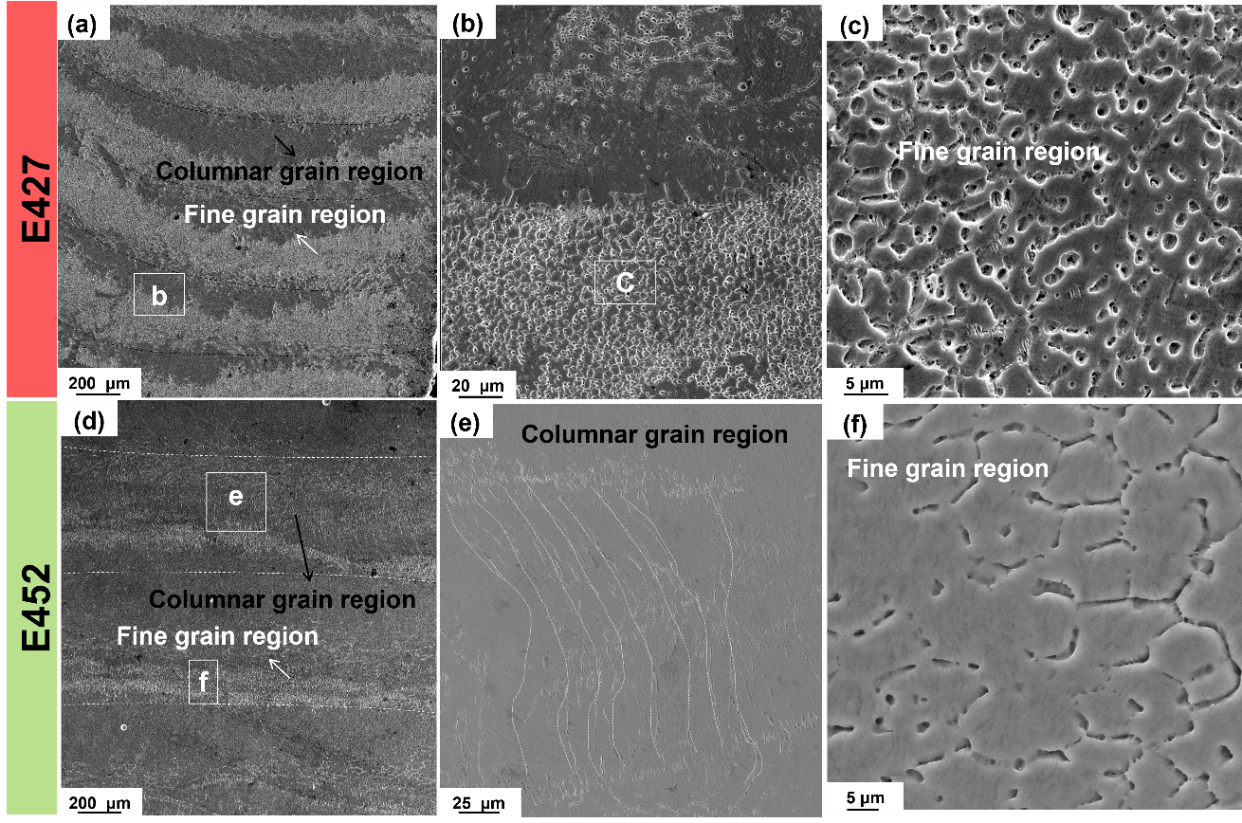


**Fig. S3** The SEM images of (a)- (c) E427 and (d)- (f) E452 NiTi samples along the building direction: (a) there are more equiaxed grains in each layer; (b) Enlarging the junction of columnar grains and equiaxed grains can be observed that (c) the grain diameter at the bottom of the layer is mostly < 10 μm; (d) The content of equiaxed grains in the E452 sample is obviously reduced; (e) The long axis of columnar grains mainly grows along the building direction; (f) The equiaxed grains whose diameter is mostly < 10 μm can be observed.


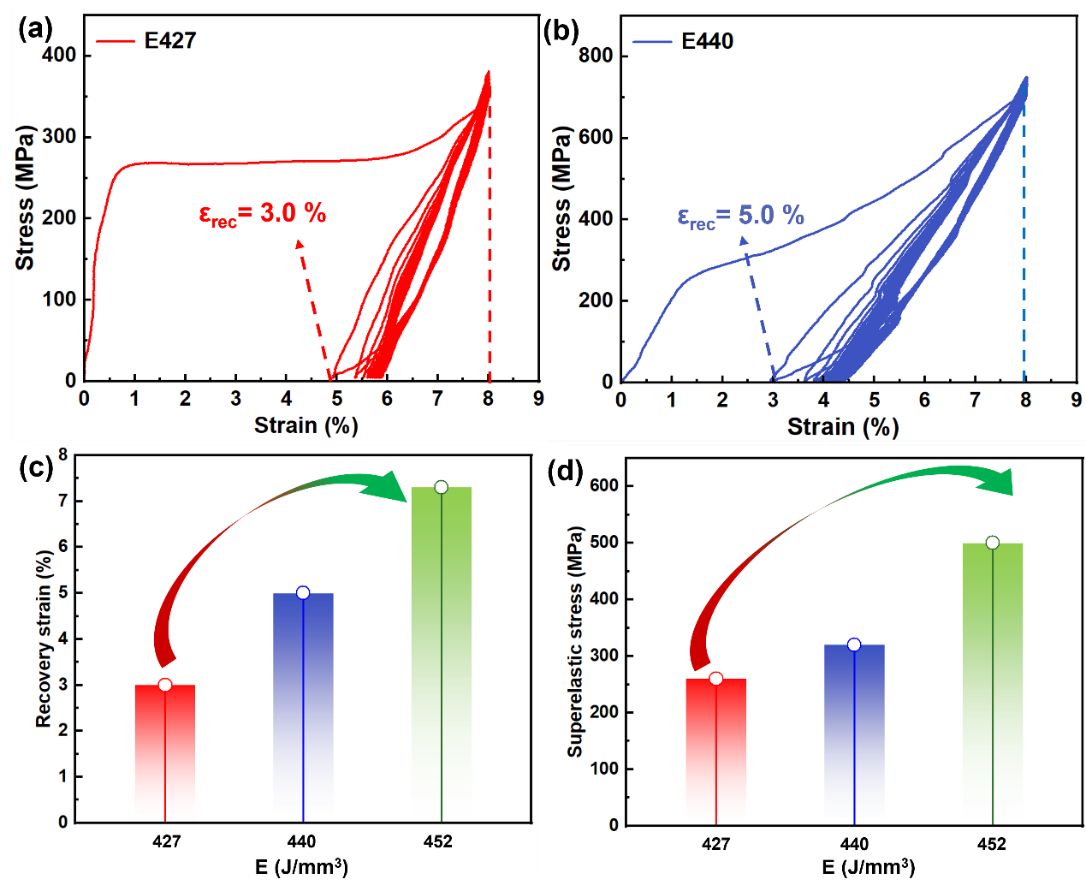


**Fig. S4** The cyclic superelastic stress-strain curves of (a) E427 and (b) E440 NiTi samples, and the recovery strains are 3.0 and 5.0 %, respectively; (c) the relationship between E and recovery strain, and (d) the superelastic stress, showing that the E452 sample with larger E owns the larger recovery strain and superelastic stress.


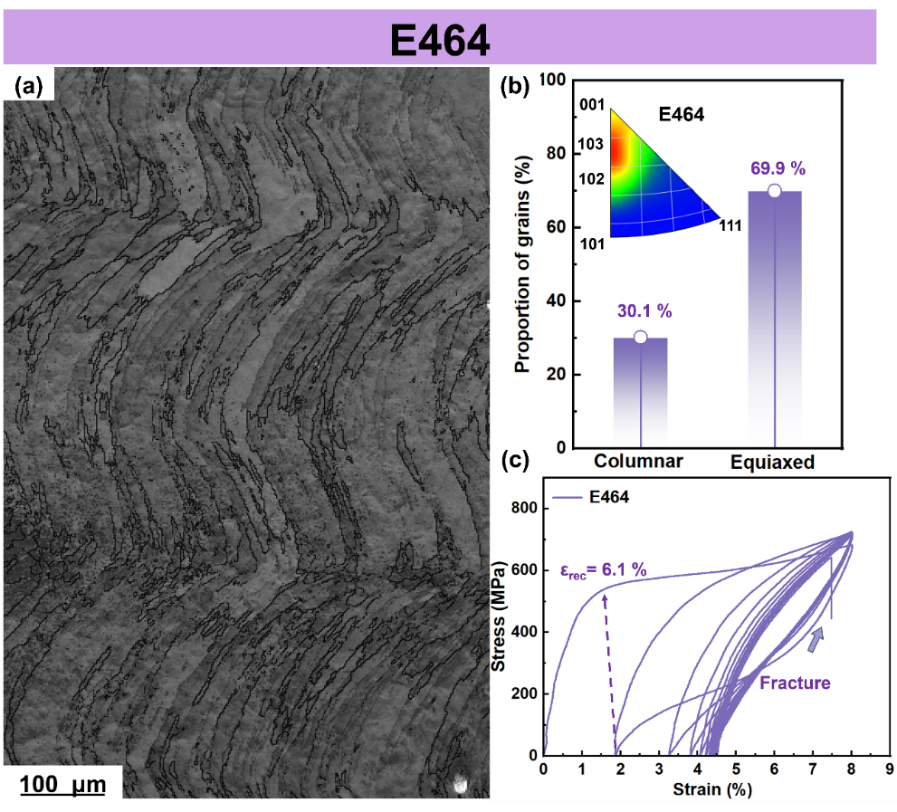


**Fig. S5** (a) The E464 sample has more columnar grains showing the stronger orientation between <102> and <103>, and (b) content of it is 30.1 %; however (c) the recovery strain is reduced to 6.1 %, and broke at the 16^th^ cycle, presenting the more columnar grains would make superelasticity worse which is not only factor to improve superelasticity of NiTi alloys.


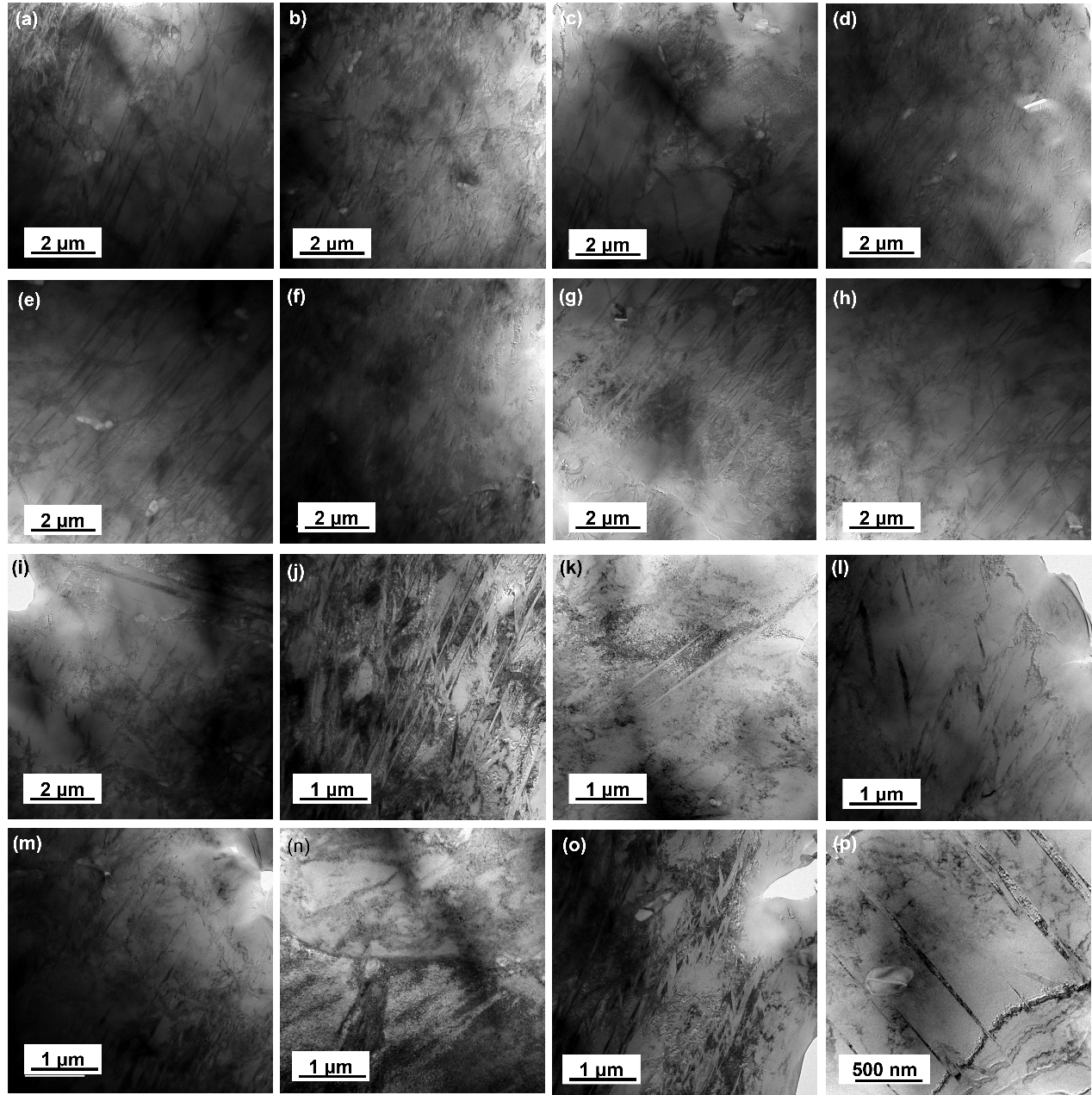


**Fig. S6** (a)-(p) The microscopic analysis of 16 random regions in the E452 sample, showing that many elongated martensite variants are distributed in the matrix, and mainly V1 variants are distributed; V2 variants can be observed in Figs. (i), (k), and (n), and V3 variants can be clearly seen in Figs. (o) and (f).


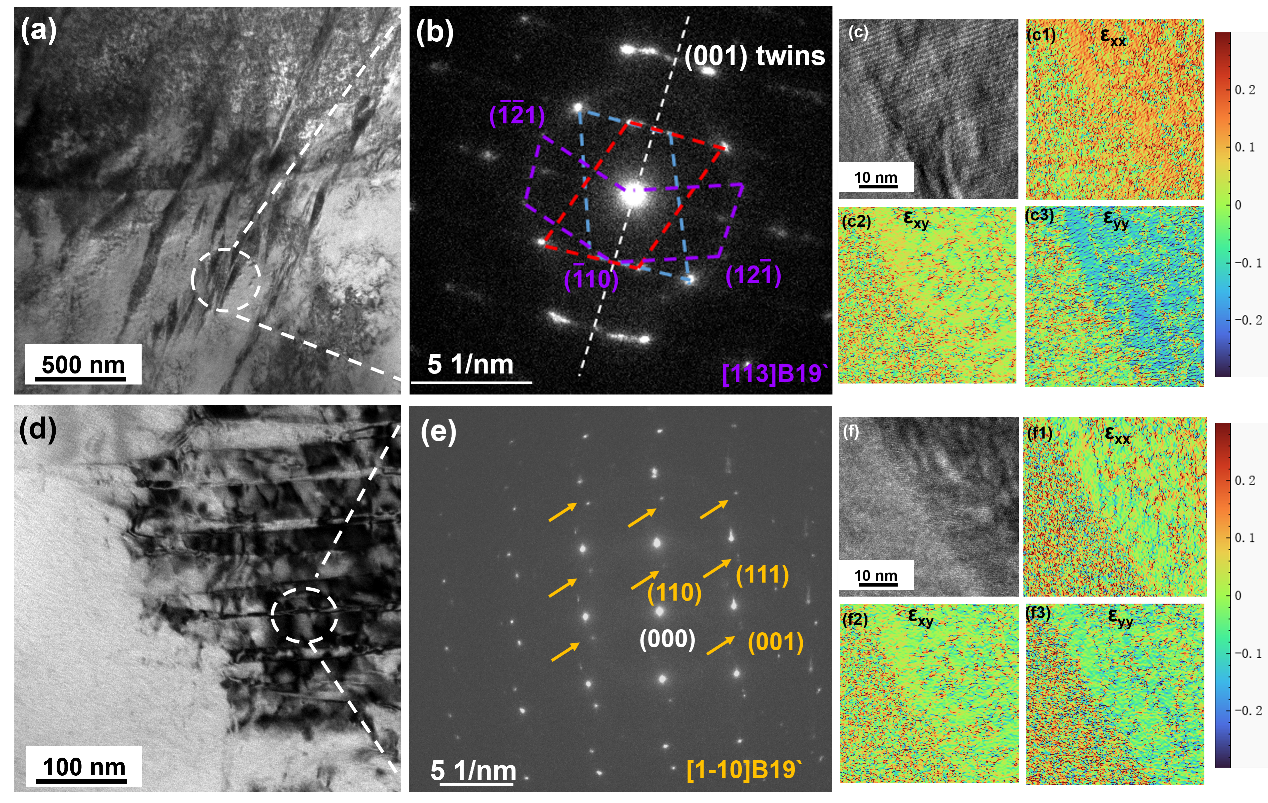


**Fig. S7** (a) V1 and (d) V2 martensite crossing the GB and (a) shorter V3 martensite; (b) The SEAD image of the area in (a) showing the different B19` phase; (c) The H-R TEM image of the interface between V3 and B2 matrix and (c1)-(c3) GPA images in different directions; (e) The SEAD image of the area in (d) showing the different B19` phase; (f) The HR TEM image of the interface between V2 and B2 matrix and (f1)-(f3) GPA images in different directions. Fig. S7 shows that the interaction between the different variants (V2 and V3) and the matrix enables phase transformation adaptation.

**
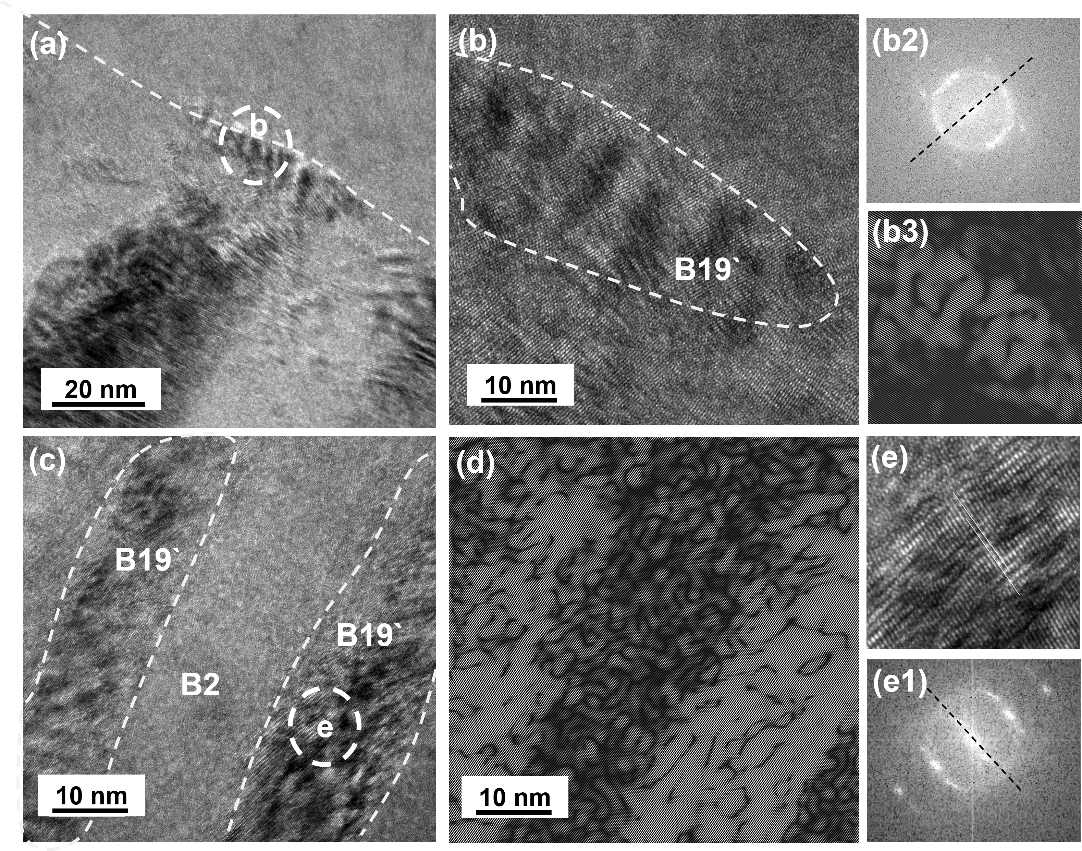
**

**Fig. S8** (a) and (b) HR-TEM images of grain boundary that prevents martensite from growing, (b) and (b1) another small martensite at the grain boundary and (b2) some dislocations with the grain boundary; (c) HR-TEM images of grain boundary through which martensite cross; (d) some dislocations between B2 and B 19`; (e) and (e1)HR-TEM image of B19` in area of (c) and (e1) its SAED diagram.


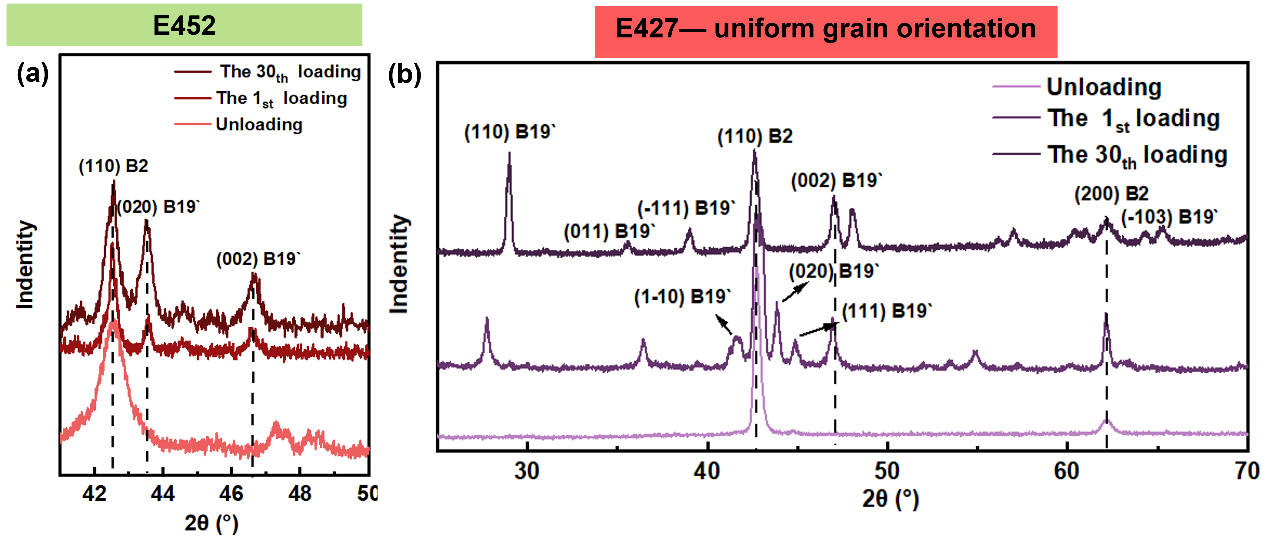
**Fig. S9** The XRD results of the (a) E452 sample and (b) E427 sample. The initial state (light line), after the 1st cycle, and after 30 cycles of loading (deep line).


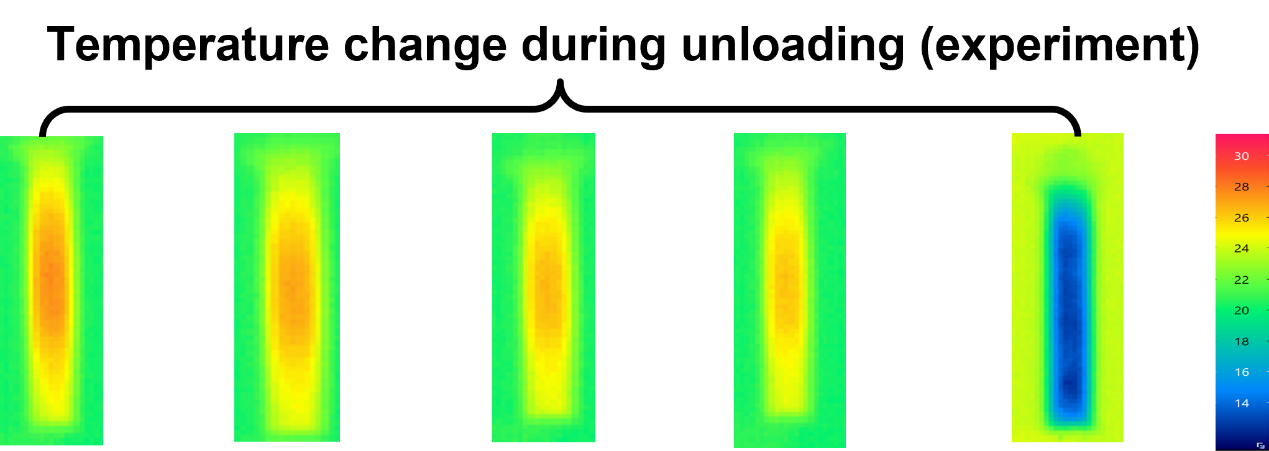


**Fig. S10** Temperature change maps of the E452 sample during unloading, showing that the reverse martensite transformation occurs uniformly, and the transformation recovery occurs in all regions at the same time, that is, it slows down to the same extent, and the central region always presents relatively high strain.


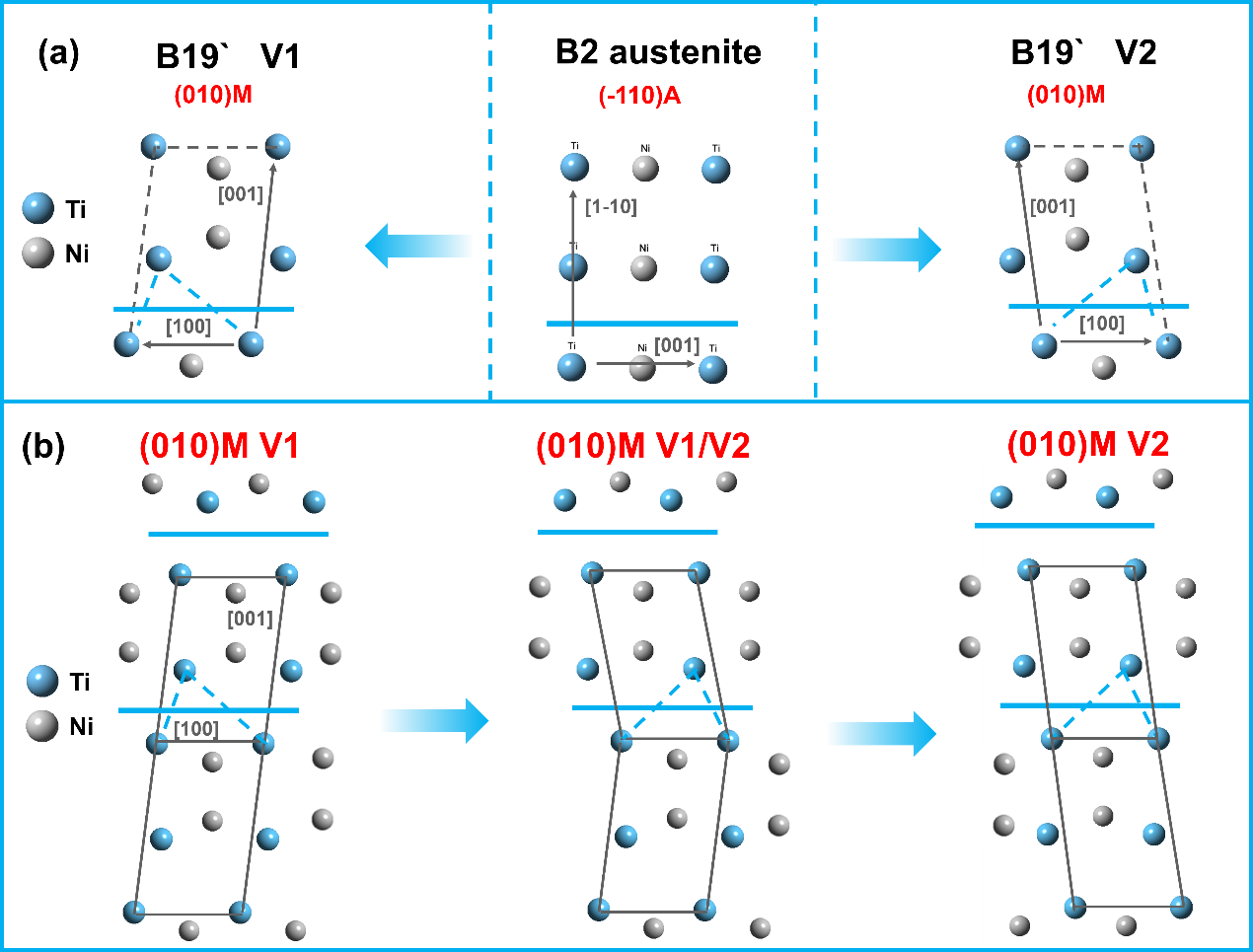


**Fig. S11** (a) The sketch of the grain structure change during the B2-B19’ martensitic transformation by showing atom positions on (-110)A cross sections along austenite planes changing into their positions on (010)M cross section along martensite planes; (b) The sketch of dislocation slip in the monoclinic B19` structure and the plastic deformation of the variant V2 by coordinated slip of partial dislocation [100](001) from V1 to V2.


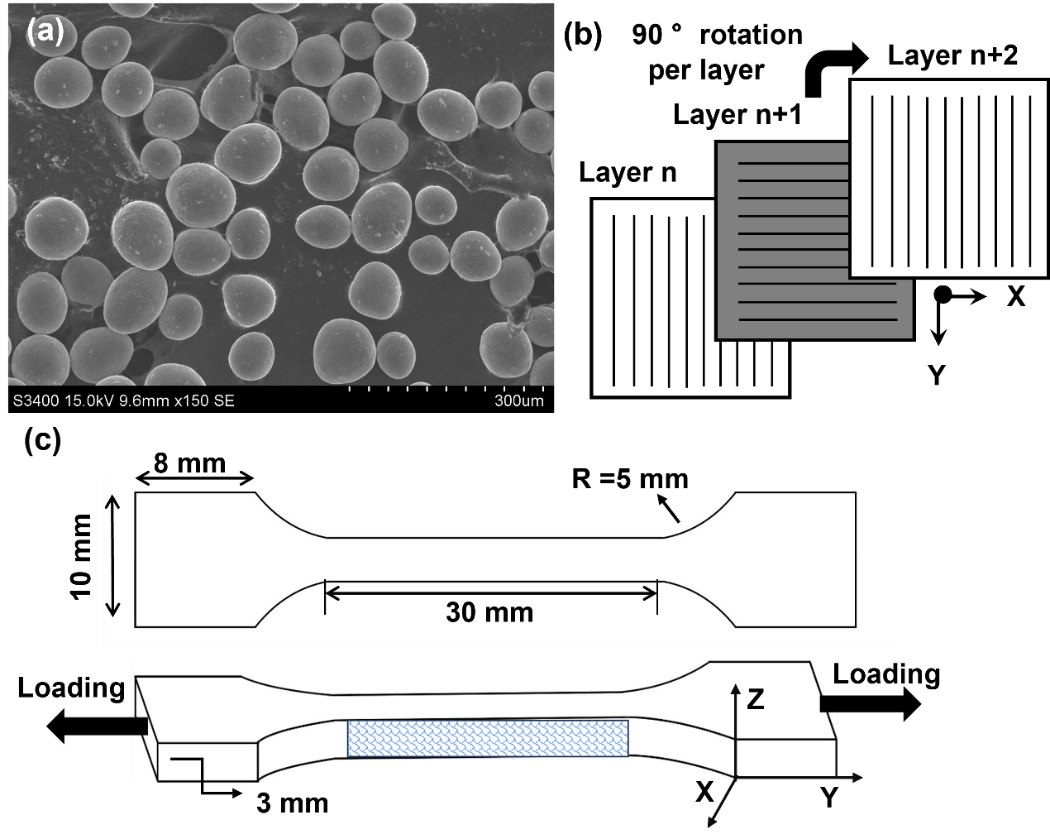


**Fig. S12** (a) The SEM image of NiTi powder, showing a uniform powder diameter; (b) Laser scanning strategy of 90 ° rotation; (c) Diagram of the tensile sample, and the shadow is the test surface of microstructure.
